# Asymmetry and rehabilitation of the subjective visual vertical in unilateral vestibular hypofunction patients

**DOI:** 10.3389/fnsys.2024.1454637

**Published:** 2024-09-10

**Authors:** Souad Haijoub, Charlotte Hautefort, Michel Toupet, Michel Lacour

**Affiliations:** ^1^Private Practitioner, Paris, France; ^2^Paris City University, Pasteur Institute, AP-HP Hôpital Lariboisière, Service ORL, INSERM, Fondation pour l’Audition, IHU reConnect, Paris, France; ^3^Centre d’Explorations Fonctionnelles Otoneurologiques, Paris, France; ^4^Department of Neurosciences, Aix-Marseille University/CNRS, Marseille, France

**Keywords:** subjective visual vertical (SVV), acute unilateral vestibular hypofunction patients (AUVP), posture, vestibular rehabilitation, tilted support protocol

## Abstract

**Aims:**

Patients with acute unilateral peripheral vestibular hypofunction (AUVP) show postural, ocular motor, and perceptive signs on the diseased side. The subjective visual vertical (SVV) test measures the perceived bias in earth-vertical orientation with a laser line in darkness. This study was aimed at (1) examining whether SVV bias could depend on preset line orientation and angles, and (2) investigating whether vestibular rehabilitation (VR) can improve SVV normalization. To our knowledge, SVV symmetry/asymmetry and impact of VR on SVV normalization have never been documented in the literature.

**Participants and methods:**

We investigated the SVV bias in a retrospective study (Study 1: *n* = 42 AUVP patients) comparing the data recorded for line orientation to the ipsilateral and contralateral sides at preset angles of 15° and 30°. We investigated the effects of VR on SVV normalization in a prospective study (Study 2: *n* = 20 AUPV patients) in which patients were tilted in the roll plane using a support tilted to the hypofunction side with the same amplitude as the SVV bias. This VR protocol was performed twice a week for 4 weeks. Supplementary data on body weight distribution and medio-lateral position of the center of foot pressure (CoP) were obtained using posturography recordings.

**Results:**

Study 1 showed asymmetrical values of the SVV bias. On average, the SVV errors were significantly higher for ipsilateral compared to contralateral line orientation (6.98° ± 3.7° vs. 4.95° ± 3.6°; *p* < 0.0001), and for 30° compared to 15° preset angle (6.76° ± 4.2° vs. 5.66° ± 3.3°; *p* < 0.0001). Study 2 showed a fast SVV normalization with VR. Non-pathological SVV bias (below ±2°) was found after only 3 to 5 VR sessions while pathological SVV values were still observed at the same time after symptoms onset in patients without VR (1.25° ± 1.46° vs. 4.32° ± 2.81°, respectively; *p* < 0.0001). A close temporal correlation was observed in the time course of body weight distribution, mediolateral CoP position, and SVV bias over time, suggesting beneficial effects of the VR protocol at both the perceptive and postural levels.

**Conclusion:**

We recommend routine assessment of the ipsilateral and contralateral SVV bias separately for a better evaluation of otolith organs imbalance that can trigger chronic instability and dizziness. The SVV bias and the postural impairment caused by the imbalanced otolith inputs after unilateral vestibular loss can be rapidly normalized by tilting the patients in the roll plane, an additional means in the physiotherapist’s toolbox. The protocol likely reweights the visual and somatosensory cues involved in the perception of verticality.

## 1 Introduction

Spatial orientation in humans requires a stable perception of the world in spite of daily life changes of the eye, head, and body position. As reviewed by Kheradmand and Winnick (2017), “orientation constancy” is a key functional aspect of spatial perception, and a prerequisite for coherent spatial perception and sensorimotor planning.

Spatial perception of verticality is a multisensory integration process based on visual, somatosensory and vestibular cues (Clemens et al., 2011). Perception of gravitational orientation integrates signals encoding eye position in the orbit, head position relative to body, and body position in space. Input from the otolith organs predominate in subjects with upright head and body position, but somatosensory cues would play a major role too (Bronstein, 1999), particularly the body sensors from the plantar sole and the head/body muscle proprioception (Guerraz et al., 1998; Yelnick et al., 2002). Contribution of orienting visual cues has also been well documented (Dichgans et al., 1975; Mittelstaedt, 1986; Kupferberg et al., 2009). The parieto-insular vestibular cortex is a cortical network involved in the perception of spatial orientation (Lopez and Blanke, 2011), in which visual, vestibular and somatosensory inputs converge (Chen et al., 2013). Lesions of the vestibular cortex affect the perception of verticality (Brandt et al., 1994), and conflicting or altered sensory cues evoke misperception of body orientation in space, spatial disorientation and impairment of gaze and postural stability. Acute unilateral peripheral vestibular pathology (AUVP) is a typical case of spatial perception disruption (Borel et al., 2001, 2008), in which dizziness and risk of falling seriously impact the patient’s quality of life.

The subjective visual vertical (SVV) test is a psychophysical task currently used to estimate the perception of verticality in both healthy subjects (Tarnutzer et al., 2009) and patients with vestibular dysfunction (Böhmer and Rickenmann, 1995). The participants are asked to report their perceived earth-vertical orientation with a laser line in total darkness or in the absence of orienting visual cues. The measurement paradigms are either based on adjustment of the laser line (actively or passively) or on a forced-choice task (see Kheradmand and Winnick, 2017). The bias estimates were similar with the two protocols but a greater variability was found with the adjustment procedure (Lim et al., 2022). The SVV values remained typically within ±2° of earth vertical in healthy participants while SVV estimates were biased towards the lesion side in patients with unilateral vestibular nerve section (10°–15°: Redon et al., 2010; Lopez et al., 2007a, 2007b) and in AUVP patients (5°–10°: Van Nechel et al., 2001; Lacour et al., 2021, 2023). The SVV bias is therefore on the same hypofunctional side as the ocular cyclotorsion, the skew deviation and the head tilt that constitute the ocular tilt reaction (Halmagyi et al., 1979). The SVV test is mainly used in clinical practice to fastly assess otolith imbalance in a relatively simple way compared to the vestibular evoked myogenic potentials (oVEMPs and cVEMPs: Clarke et al., 2005).

Normalization of the SVV over time, i.e., return to SVV estimates in the non pathological range (< ± 2°), is a compensation process that takes time. The literature showed that the SVV bias was reduced but still present 3 months after symptoms onset in AUVP patients, and that 6 months to 1 year were often necessary for full spontaneous SVV normalization (Curthoys and Halmagyi, 2014). Vestibular rehabilitation therapy (VR) is commonly used by physiotherapists to improve the patient’s quality of life, and to speed up the compensation process. The Cochran database of systematic reviews indicate that there is moderate to strong evidence that VR is a safe, effective management for unilateral peripheral vestibular dysfunction, based on a number of high quality randomized controlled trials (Hall et al., 2022). There is currently no systematic study on the effect of specific VR protocols on SVV normalization with session after session monitoring and, to our knowledge, how VR can improve SVV normalization has never been investigated. We have recently reported asymmetrical SVV bias values in a limited population of AUVP patients, with higher estimates for ipsilateral compared to contralateral line orientation that had never been documented in the literature (Haijoub and Lacour, 2024). Clinical SVV investigations generally measure the mean bias from data collected independently of the side of the laser line orientation (ipsilateral or contralateral with respect to the diseased side), and independently of the preset angle of line orientation (15°, 30° for example).

**Figure 1:**
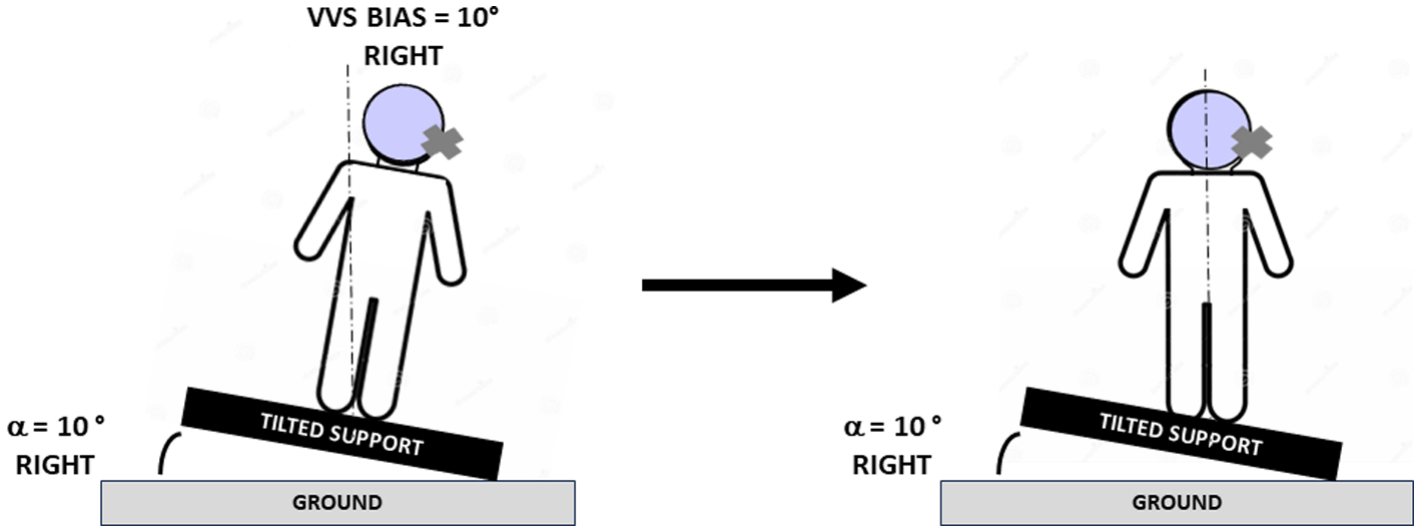
Protocol for rehabilitation of the subjective visual vertical. Schematic drawings illustrating the protocol used to normalize the subjective visual vertical (SVV) and the posture of the patients. The pictures show a patient with acute unilateral vestibular hypofunction on the right side and a SVV bias of 10° on the right side at the beginning (left) and at the end (right) of the rehabilitation session. The patient was tilted in the roll plane by using a support reproducing an inclination of 10° to the same side as the vestibular deficit, and he/she was asked to stand quietly on the tilted support, first with eyes open, then with eyes closed (see text). Body re-orientation in space with postural corrections consisting of body displacement to the opposite healthy side were observed during the rehabilitation session. The dashed line indicates the gravitational vertical.

The study was first aimed at determining in a larger sample of AUVP patients the factors responsible for the SVV asymmetrical bias. This was done in a retrospective study investigating the ipsilateral and contralateral SVV bias in 42 acute patients tested in the 2–14 days time window after symptoms onset. A second prospective study was designed to analyze the normalization of the SVV in 20 AUVP patients submitted to SVV rehabilitation with the tilted support protocol. This original protocol was proposed by French physiotherapists some years ago as a way to accelerate SVV normalization. It consists in tilting the base of support on which patients were asked to stand and keep balance, to the hypofunction side, with a tilt angle of the same amplitude as the SVV bias, in order to produce postural corrections in the opposite direction compared to the postural and perceptive deficits (Figure 1). Whether the protocol works or not has never been validated, no results have been published, and the underlying mechanisms by which it could reduce the SVV bias remain largely unknown. Normalization of the SVV over time was analyzed together with posturography recordings of the medio-lateral position of the center of foot pressure and of the distribution of body weight on each leg.

## 2 Participants and methods

### 2.1 Participants

Neurotological examination of the AUVP patients was done in one tertiary referral center (Centre d’Explorations Fonctionnelles Otoneurologiques, Dr. Toupet, Paris) and in one ENT Department (Hôpital Lariboisière, Dr. Hautefort, Paris) where the patients were diagnosed. All AUVP patients exhibited the five main inclusion criteria proposed by Strupp and Magnusson (2015): acute onset of spinning vertigo, horizontal rotatory spontaneous nystagmus (SN) beating to the intact side, a positive head impulse test (HIT) on the weaker side, nausea, and postural imbalance.

Angular vestibulo-ocular reflex (aVOR) gains recorded during passive video head impulse test (vHIT Otometrics) below 0.70 and presence of overt/covert saccades were used to determine the pathological weaker side. Horizontal aVOR gain on the intact side above 0.80 was also required for patients’ inclusion. Positional vertigo, central vestibular pathology, ocular motor dysfunctions, and drug treatment were exclusion criteria. Vestibular deficit was documented on the basis of the HIT for the lateral, anterior and posterior canals. Caloric vestibular testing was not systematically done due to discomfort, but was always pathological on the weaker side when performed. The ocular and cervical vestibular evoked myogenic potentials (oVEMPs and cVEMPs) were not systematically investigated. All patients provided written informed consent to participate and were asked to abstain from antivertigo drugs for the duration of the study.

### 2.2 Study 1: asymmetry of the SVV bias

Study 1 is a retrospective analysis focused on the SVV bias estimates recorded with the SVV adjustment protocol and laser line orientation either to the ipsilateral weaker side or to the contralateral healthy side, at preset angles of ±15° and ± 30°. The study population comprised 42 patients, 24 females and 18 males with a mean age of 56.6 ± 17.6 years, whose initial visit took place on average 7.8 ± 3.3 days after symptoms onset. The initial visit was the day of study inclusion. The weaker side was the left for 27 patients and the right for 15 patients (see Table 1 for patients’ characteristics).

**Table 1:** Characteristics of the populations of patients with acute unilateral vestibular pathology.

	Retrospective study 1 (*N* = 42)	Prospective study 2 (*N* = 20)	Controls (*N* = 39)
Disease side	27 Left	13 Left	24 Left
	15 Right	7 Right	15 Right
Sex	24 Females	14 Females	22 Females
	18 Males	6 Males	17 Males
Age (mean and SD in years)	56.6 ± 17.6	47.6 ± 19.7	62.1 ± 14.7
(Range)	(24–86)	(22–87)	(25–86)
Time from symptoms onset (mean and SD in days)	7.8 ± 3.3	8.4 ± 5.7	27.3 ± 9.7
(Range in days)	(2–14)	(2–14)	(18–35)
Ipsilateral horizontal aVOR gain	0.35 ± 0.21	0.29 ± 0.17	0.26 ± 0.12

Mean values are presented with the standard deviation and range for age of the patients (in years), time from symptoms onset (in days), or with numbers only for sex and disease side. Mean values and standard deviations of the angular vestibulo-ocular reflex gain recorded during the head impulse test (HIT) are shown for HIT performed on the horizontal semicircular canal on the weaker side. Data are shown for the retrospective study 1 (left column), the prospective study 2 (middle column), and the controls (right column).

### 2.3 Study 2: normalization of the SVV with vestibular rehabilitation

Study 2 is a prospective analysis of the SVV normalization investigated in AUVP patients who received vestibular rehabilitation with the tilted support protocol. The study population comprised 20 patients, 14 females and 6 males with a mean age of 47.6 ± 19.7 years, whose initial visit took place early after symptoms onset (8.4 ± 5.7 days on average). The initial visit was the day of study inclusion and the day of the first rehabilitation session. The effect of VR was assessed by comparing the SVV biases recorded at the beginning of each rehabilitation session for line orientation towards either the ipsilateral weaker side or the contralateral healthy side. Thirty nine AUVP patients without VR constituted the control group. Assessments of the control patients were done later compared to the patients receiving the VR protocol (27.3 ± 9.7 days) due to late vestibular neuritis diagnosis. The only rehabilitation the controls could have had resulted from their own activity at home, generally limited by their fear of falling and limitations of head movements inducing oscillopsia (see Table 1 for patients’ characteristics). Study 2 included supplementary posturography measurements of the mediolateral position of the center of foot pressure (CoP) and of the body weight distribution on each leg as a function of the VR sessions.

### 2.4 Assessment of vestibular deficit

HIT was manually imposed and performed with passive head rotation to the healthy and weaker sides in seated patients. Head rotations were done with ∼10° peak amplitude, ∼200°/s peak velocity and ∼2,000°/s^2^ peak acceleration. Recording of the aVOR for the horizontal canals was done by tilting the patient’s head downwards by 30° to place the lateral semicircular canals in the horizontal plane. Recordings of the aVOR of the anterior and posterior canals were done by turning the patient’s head 45° to the right and to the left. HIT was performed randomly to elicit unpredictable timing and direction of head movement. The aVOR gain values were approximated by the Otometrics software as the ratio peak eye velocity/peak head velocity. An average gain value was calculated from 5 correctly performed tests on the intact and weaker sides. However, more than 5 trials were generally done due to blinks or imperfect target fixation.

### 2.5 Assessment of the subjective visual vertical

Experiments were carried out with participants standing and facing a screen 1.5 meter away in front of them. Their arms were positioned along the body in a natural way. Participants wore goggles narrowing the visual field and suppressing all the orienting visual cues. The SVV perception was assessed using the adjustment protocol. A red laser line was projected on the screen (Framiral, Cannes, France). The line orientation was either towards the ipsilateral weaker side or towards the contralateral healthy side, with preset angles of ±15° and ±30° pseudo-randomly distributed. The starting position of the laser line was to the right, i.e., ipsilaterally for the patients with right vestibular hypofunction and contralaterally for those with left vestibular hypofunction. By convention, SVV bias values were positive for line orientation to the hypofunction side. The line was automatically rotated clockwise or counterclockwise at low velocity (2°/s), and the participants were instructed to align the laser line with their perception of verticality by verbal response. They had to say “stop” when they perceived the line orientation as being vertical. SVV judgment was performed binocularly from a total number of 10 trials for all the conditions (2 sides and 2 angles), with the participants’ head totally free and upright. The mean values were calculated independently for the ipsilateral and contralateral line orientations, and for the 2 preset angles.

### 2.6 Vestibular rehabilitation with the tilted support protocol

The rationale for the protocol is to produce a body tilt in the direction of the SVV bias so that the patients correct their posture by moving to the opposite side, in the direction opposite to their perceived vertical. The patients were asked to stand quietly on a platform inclined to the same side as the SVV bias and with the same amplitude. The protocol was first performed with eyes open. The patients showed postural instability and body tilt to the side of platform tilt, and then corrected their posture by displacing their CoP to the opposite side, using both body sensors and orienting visual references. Once the patients were less unstable and had more or less restored an upright posture, the test was performed with the eyes closed. In this visual condition, the information from plantar sole receptors and leg muscle proprioception was predominant to keep balance on the tilted platform. In a final step, the patients were trained to keep balance with eyes open and eyes closed on foam placed on the inclined support. The protocol duration was around 20 min: 5 min in each visual condition on the tilted support, and 5 min in each visual condition on foam. Time spent in each condition could however vary depending on the ability of the patients to keep balance. Adjustments were necessary to avoid disequilibrium and falls that could have compromised the patients’ participation in the following VR sessions. VR was done twice a week for 4 weeks after inclusion, with the same physiotherapist (SH). It was stopped when the ipsilateral and contralateral SVV estimates regained non-pathological values, i.e., below 2°, during 3 consecutive VR sessions.

A typical VR session lasted 30 min on average. It began with measurement of the SVV bias and the posturography investigation, followed by rehabilitation on the tilted support, and re-testing of SVV and posture. VR efficacy was assessed by comparing the data collected in one session to those recorded at the beginning of the following session. All the AUVP patients normalized their SVV after 5 VR sessions, that is, within 30 days after symptoms onset. The SVV data collected ~30 days after symptoms onset in AUVP patients without rehabilitation (*N* = 39) were used as a control group.

The experimental protocol followed the recommendations of the Declaration of Helsinki, and informed consent was obtained from each patient before participation.

### 2.7 Posturography measurements

Posturography investigation was performed with the “Cyber sabots” posturography platform (In Tech, Italia), made of two separate and independent dynamometric platforms, one for each foot, 30° apart. Body sway was recorded on the two stable force plates sensing the vertical force on the ground by means of 3 strain-gauge force transducers providing description of body sway in terms of displacement of the center of foot pressure (CoP) in the anteroposterior and medial-lateral directions. One innovative feature of the platform was to give access to separate analysis of the stabilogram and to body weight distribution on each leg. Posturography data were recorded at a sampling frequency of 40 Hz (16 bits) with eyes open (EO) during 55 s and with eyes closed (EC) for the same time period. The patients were required to stand quietly without voluntary movements of head and body. Body sway was assessed by computing the medio-lateral position of the CoP over time (in mm), and by measuring the sway area contained in the confidence ellipse including 95% of the CoP positions sampled (in mm^2^) in each visual condition. The software also provided the body weight distribution on each leg in percent of the total body weight and the Romberg ratio on both sway length and sway velocity defined as [(EC score − EO score)/(EC score + EO score) × 100]. These ratios quantify the influence of vision on postural control and the energy spent to keep balance, respectively. The total body weight being 100%, it is expected to be equally distributed on the right (~50%) and left (~50%) legs in healthy participants during quiet standing, and to show asymmetrical body weight distribution in AUVP patients with higher percentage on the ipsilateral leg as the result of vestibular tone imbalance in the roll plane.

### 2.8 Statistical analysis

Statistical analysis was carried out using repeated-measurement analyses of variance (ANOVAs). The SAS Proc Mixed repeated-measurement procedure was used to assess the vestibular rehabilitation-induced changes of the SVV bias over time (Littel et al., 1996). This analysis incorporated three within-subjects factors: the VR sessions (sessions 1, 2, 3, 4, and 5), the direction of the laser line orientation (ipsilateral and contralateral), and the preset angle (± 15° and ± 30°). Efficacy of VR on the SVV normalization was done by analyzing the SVV bias as a function of the training sessions on the tilted support, and by comparing the data recorded at the end of VR to those recorded at a similar time period after symptoms onset (1 month) in a control group of AUVP patients without VR on the tilted support.

Similar ANOVAs were performed on the two posturography parameters recorded in our AUVP patients: the medio-lateral position of the CoP and the body weight distribution on the ipsilateral and contralateral leg with respect to the side of the vestibular loss. *Post hoc* analysis were done with the Scheffe test and the multicomparison Fisher test (Statview II software).

## 3 Results

### 3.1 Study 1: asymmetry of the SVV estimates

The ANOVA performed on the SVV bias recorded in the 42 AUVP patients evidenced significant effects of the two levels of line orientation [*F*_(1,334)_ = 68.4, *p* < 0.000004] and the four levels of preset angle amplitude [*F*_(3,334)_ = 37.6, *p* < 0.0001]. *Post hoc* analyses showed ipsilateral SVV estimate significantly higher with line orientation to the ipsilateral side compared to line orientation to the contralateral side (*p* < 0.0001), and at 30° compared to 15° preset angle (*p* < 0.0001).

**Figure 2:**
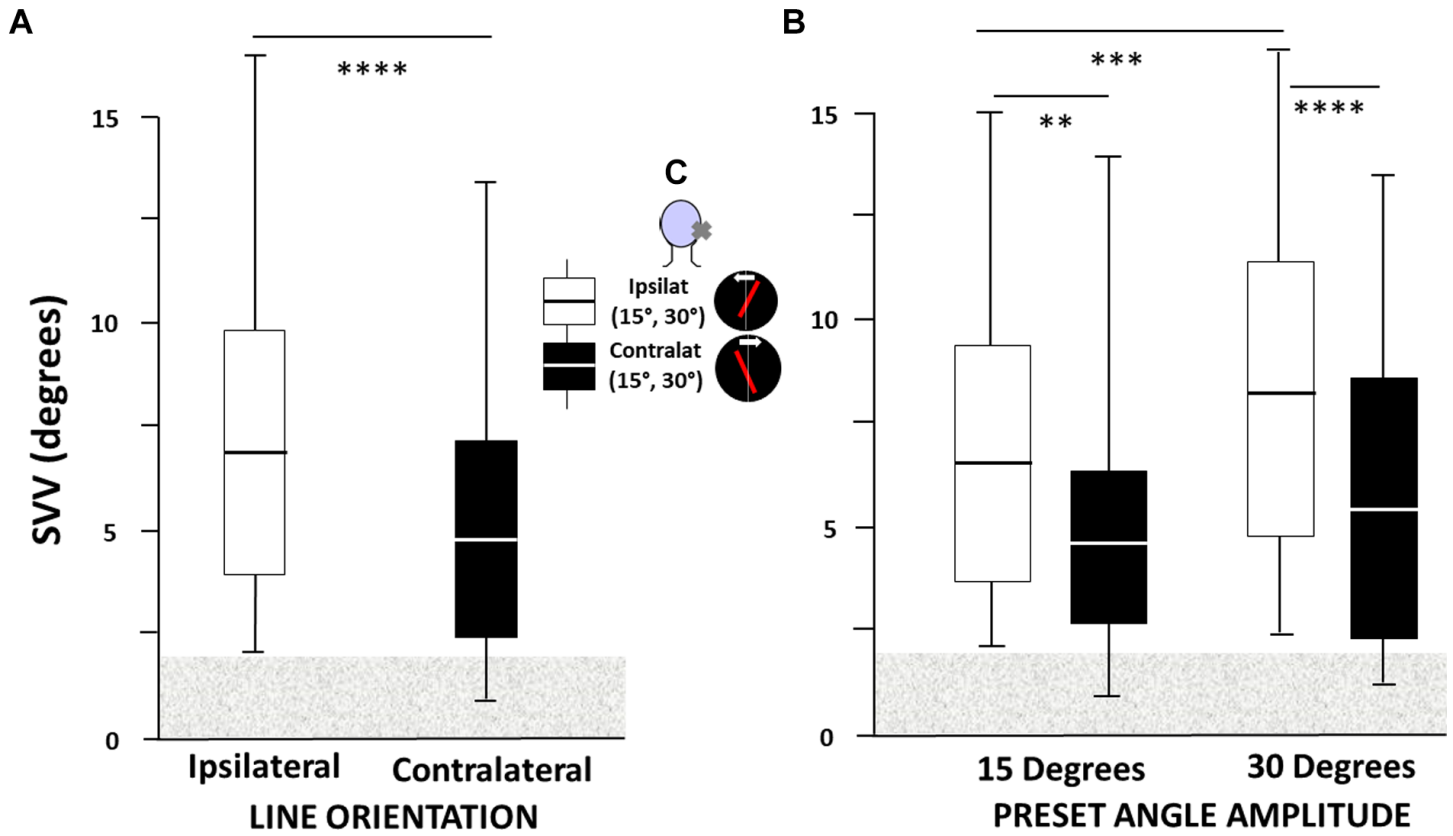
**(A–C)** Asymmetry of the subjective visual vertical (SVV) bias in acute unilateral peripheral vestibular hypofunction patients depends on both laser line orientation and preset angle. All patients had SVV bias on the same side as the vestibular deficit regardless of the ipsilateral or contralateral orientation of the laser line and, by convention, positive SVV bias was to the same side as the vestibular deficit for both patients with right-or left-sided deficits. **(A)** The SVV bias (in degrees) is significantly higher when the laser line is initially oriented to the side of the vestibular hypofunction (ipsilateral) compared to line orientation to the healthy side (contralateral), and **(B)** at 30° compared to 15° preset angle. The boxplots in A were elaborated independently of the preset angle value while in **B** they took into account both line orientation and preset angle amplitude. Boxplots show the median with the first and third quartiles, and whiskers indicate the minimum and maximum SVV values. The horizontal grey area indicates the non-pathological SVV range. ^**^p < 0.005, ^***^p < 0.001, and ^****^p < 0.0001. The inset in **C** summarizes the SVV protocol, with the laser line orientation (red tilted bar) oriented ipsilaterally or contralaterally with respect to the hypofunction side (cross on the patient’s head), at preset angles of 15° or 30°, and rotating to the healthy or weaker sides at slow velocity (2°/sec: white arrow).

Figure 2C shows an insert summarizing the experimental protocol, with the laser line oriented ipsilaterally or contralaterally with respect to the disease side, at preset angles of 15° or 30°, and line rotation in the opposite direction. The boxplots illustrate the 1st and 3rd quartiles of the SVV estimates, with the median, and whiskers indicate the minimum and maximum values, as a function of line orientation (Figure 2A) and preset angle amplitude (Figure 2B). By convention, SVV biases to the same side as the vestibular deficit were rated positively for both patients with right-or left-sided deficits. The results showed that the SVV bias was always on the same side as the vestibular deficit regardless of the ipsilateral or contralateral orientation of the laser line. They also showed asymmetrical SVV biases, with significantly higher bias for ipsilateral compared to contralateral line orientation (6.98° ± 3.7° vs. 4.95° ± 3.6°, respectively; *p* < 0.004), and significantly higher bias at preset angle of 30° compared to 15° (8.18° ± 4.1° vs. 6.36° ± 3.29°, respectively; *p* < 0.004). The contralateral SVV bias did not show a preset angle effect (5.23° ± 3.90° vs. 4.90° ± 3.40° for the 30° and 15° angles, respectively; *p* = 0.59). The highest mean SVV bias was found with ipsilateral line orientation and 30° preset angle.

### 3.2 Study 2: rehabilitation with the tilted support protocol

#### 3.2.1 Effects on the SVV estimates

The ANOVAs with mixed models were conducted on the ipsilateral and contralateral SVV estimates of the 20 AUVP patients as a function of the VR sessions. They evidenced significant effects of VR session [*F*_(4,78)_ = 78.32, *p* < 0.01] and of line orientation [*F*_(1,39)_ = 96.74, *p* < 0.0001]. The ANOVA also indicated that session was the main effect providing the sources of variation, and *post hoc* analyses showed only significant differences between session 1 and 2 (*p* < 0.01), and between sessions 2 and 3 (*p* < 0.05). In addition, a significant interaction of session × line orientation was observed for both ipsilateral and contralateral line orientations [*F*_(4,78)_ = 36.70, *p* < 0.01].

**Figure 3:**
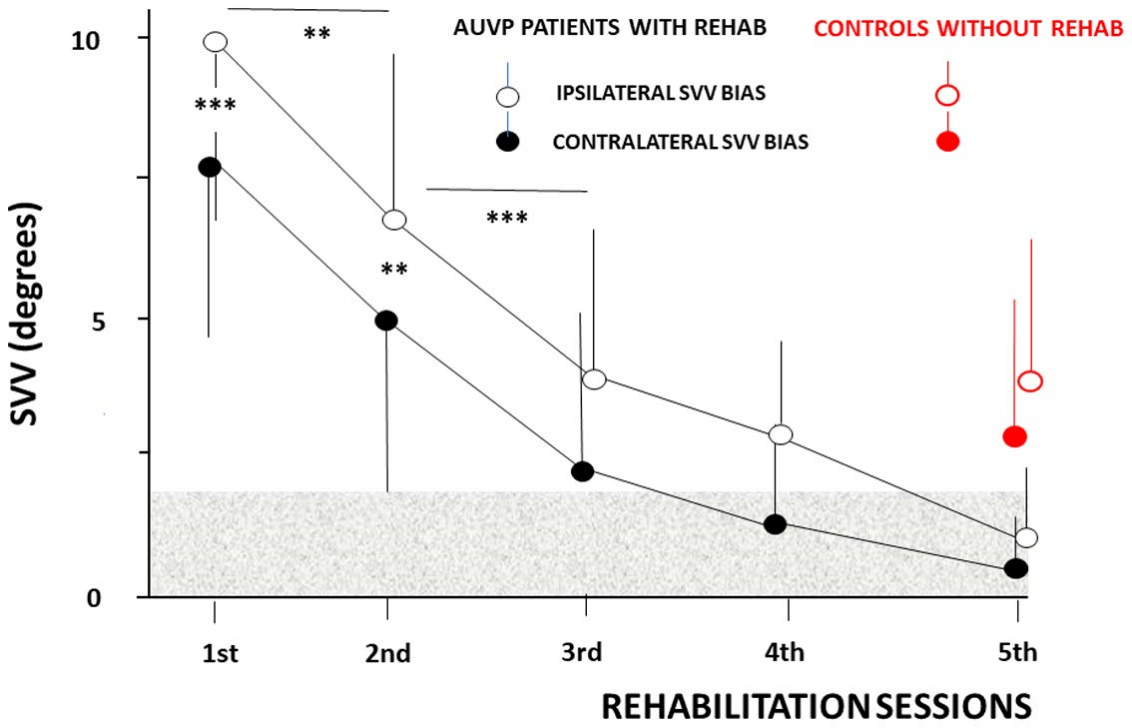
Normalization of the subjective visual vertical (SVV) as a function of the rehabilitation sessions in patients tilted in the roll plane. The ipsilateral (empty circles) and contralateral (filled circles) mean SVV biases (ordinates, in degrees) are plotted with their standard deviation (vertical thin solid lines) as a function of the rehabilitation sessions (abscissae). The ipsilateral and contralateral mean SVV values differ significantly during the first two sessions, with higher bias for ipsilateral line orientation, and they progressively reduce with repetition of the rehabilitation sessions (same convention for the SVV bias as in Figure 2). The horizontal grey area indicates the non-pathological SVV range. SVV estimates recorded at the same time period after symptoms onset (around 1 month) in patients without vestibular rehabilitation are plotted for comparison (red filled and empty circles). ^**^p < 0.005 and ^***^p < 0.001.

Figure 3 illustrates the mean SVV bias recorded at the beginning of each VR session. The SVV was systematically tilted towards the weaker side at the inclusion visit, regardless of the ipsilateral or contralateral orientation of the laser line, and averaged 9.98° ± 4.20° and 8.02° ± 4.57° for the ipsilateral and contralateral SVV estimates, respectively (*p* < 0.001). *Post hoc* analyses showed a significant reduction of the SVV bias as early as the second VR session (6.97° ± 2.94° vs. 5.21° ± 3.25°, ipsilaterally and contralaterally, respectively; *p* < 0.005), and at the third VR session (3.96° ± 2.71° vs. 2.27° ± 2.97°, ipsilaterally and contralaterally, respectively; *p* < 0.001). Later on, the SVV bias progressively normalized. However, the contralateral SVV bias was normalyzed earlier, at the fourth VR session (1.51° ± 2.13°) compared to the ipsilateral SVV estimate that still remained pathological at this moment (3.23° ± 2.16°). This observation justifies working separately on the ipsilateral and contralateral values, and not on the overall score which can lead to wrong interpretation regarding SVV normalization. All the AUVP patients normalyzed their SVV at the fifth VR session, that is around 1 month after their symptoms onset (1.25° ± 1.46 °vs. 0.47° ± 1.21° for the ipsilateral and contralateral SVV bias, respectively). At this time period, the control group of AUVP patients without VR still exhibited significantly higher ipsilateral (4.32° ± 2.81°) and contralateral (3.20° ± 2.80°) SVV estimates compared to our population of AUVP patients with VR (*p* < 0.0001 and *p* < 0.0002, respectively).

#### 3.2.2 Effects on posture control

The ANOVA with mixed models performed on the CoP parameter evidenced significant effects of VR session [*F*_(4,78)_ = 37.4, *p* < 0.001]. Session was again the main effect providing the sources of variation, and *post hoc* analyses only showed significant differences between the first two sessions (*p* < 0.005). In addition, a significant interaction of session × visual condition × medio-lateral CoP position was observed [*F*_(4,78)_ = 36.70, *p* < 0.01]. The ANOVA also confirmed significant differences between the EO and EC conditions during the first two VR sessions.

**Figure 4:**
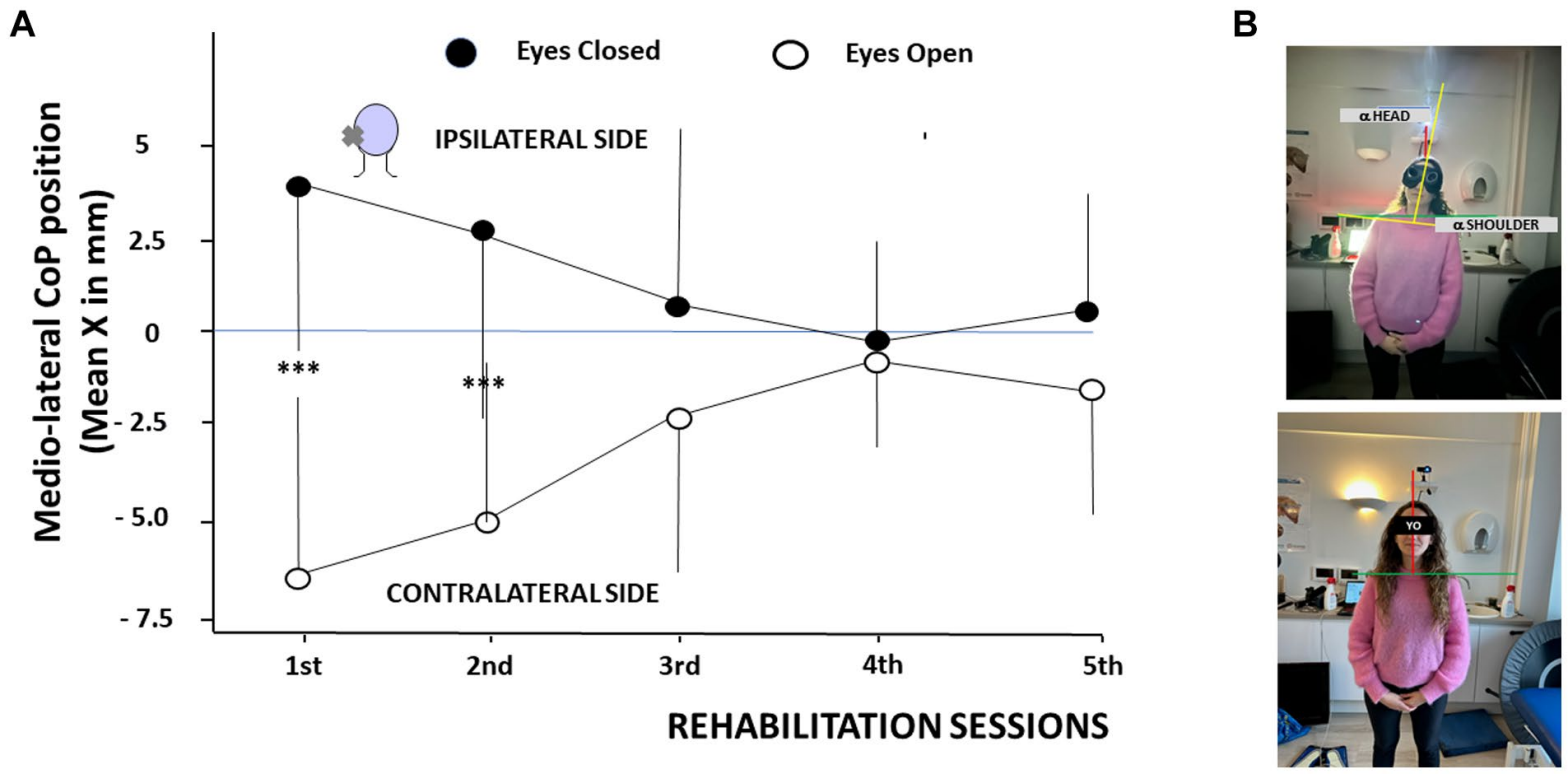
**(A,B)** Modifications of the center of foot pressure as a function of the rehabilitation sessions in patients tilted in the roll plane. **(A)** Changes in the mean medio-lateral position of the center of foot pressure (CoP, ordinates, in mm, with SD) as a function of the rehabilitation sessions (abscissae). By convention, positive values indicate a shift of the CoP towards the hypofunction side (ipsilateral side), and negative values to the healthy side (contralateral side). The posturography recordings show a CoP deviation to the affected side with the eyes closed (filled circles) and to the healthy side with the eyes open (open circles) during the first two sessions. CoP medio-lateral position normalizes later on. ^***^p < 0.001. **(B)** Illustration of the head and trunk orientation in a patient with acute unilateral vestibular hypofunction on the left side examined 2 days after symptoms onset without orienting visual cues (upper photo) and with vision (lower photo). Note the head/trunk deviation to the hypofunction side without vision and an immediate posture correction with orienting visual cues.

Figure 4A illustrates the mean medio-lateral position of the CoP recorded as a function of the VR sessions and of the visual condition. By convention, positive values indicated a CoP deviation towards the ipsilateral hypofunction side, and negative values towards the contralateral healthy side. Posturography recordings were performed at the beginning of each VR session in the eyes open (EO) and eyes closed (EC) conditions. The data showed CoP deviations to the ipsilateral side with EC, and to the opposite contralateral side with EO during the first two VR sessions. The mean CoP values were + 3.53 mm ± 4.7 mm and − 6.53 mm ± 3.5 mm at the first VR session in EC and EO conditions, respectively (*p* < 0.0003), and + 2.74 mm ± 4.5 mm and − 5.2 mm ± 5.1 mm at the second VR session with EC and EO, respectively (*p* < 0.0007). Later on, the CoP position did not differ significantly between the two visual conditions and was close to zero at the 4th and 5th VR sessions. The data strongly suggest a major contribution of the orienting visual cues on the medio-lateral position of the CoP soon after symptoms onset. This is illustrated in Figure 4B in a patient tested 2 days after a left vestibular neuritis without (upper photo) and with (lower photo) vision. Head and trunk deviations (α head angle and α shoulder angle) were close to zero when orienting visual cues were present.

**Figure 5:**
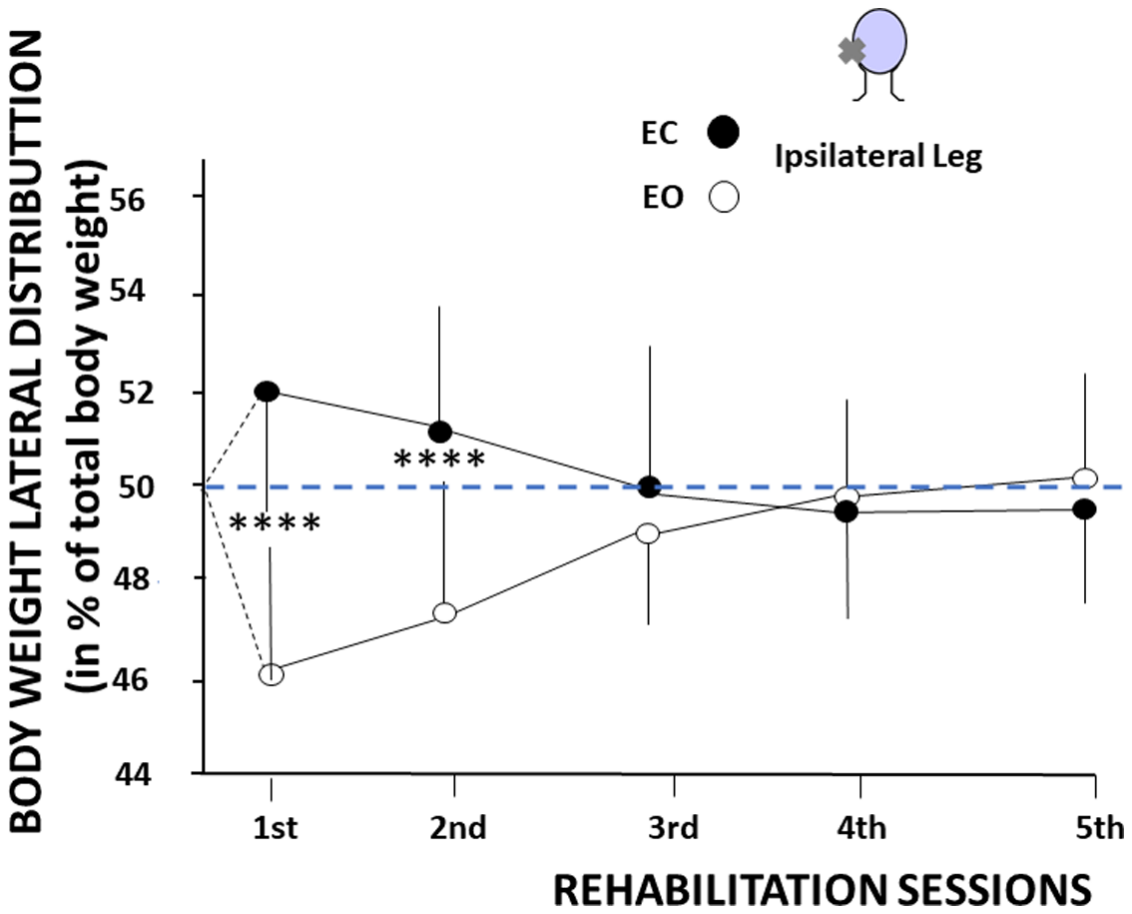
Changes in body weight distribution as a function of the visual context and the rehabilitation sessions. The figure shows the body weight distribution on the ipsilateral leg, expressed in percent of total body weight (ordinates) as a function of the rehabilitation sessions (abscissae). Mean values are plotted in the eyes open (open symbols) and eyes closed (filled symbols) conditions with their standard deviation (thin vertical line). The figure shows a higher body weight distribution on the leg ipsilateral to the vestibular deficit with eyes closed, and a lower body weight distribution on the same leg with eyes open. An opposite pattern was recorded on the contralateral leg.

The same recovery pattern was observed for the body weight distribution. Figure 5 illustrates the shift in the body weight distribution recorded on the ipsilateral leg, on the weaker vestibular side, as a function of the visual condition. The body weight distribution was significantly higher on the ipsilateral leg with EC, compared to the lower weight distribution observed on the same leg in the EO condition. The total body weight being 100%, an opposite pattern was observed on the contralateral leg (not illustrated here). This finding was only observed during the first two VR sessions, confirming the modifications of the medio-lateral CoP position described in Figure 4A. The mean body weight distribution at the first VR session shifted from 51.9% ± 3.4% on the ipsilateral leg with EC to 46.3% ± 2.9% on the same ipsilateral leg with EO (*p* < 0.0001), and from 51.1% ± 3.4% ipsilaterally with EC to 47.2% ± 2.8% ipsilaterally with EO (*p* < 0.0001) at the second VR session. Later on, the body weight was symmetrically distributed on both legs in both visual conditions.

The posturography analysis also pointed to changes in the Romberg ratios assessed on the surface and the velocity of the CoP displacements. Results showed reduced Romberg ratios from the first to the last VR sessions. Table 2 illustrates the mean values (± SD) of the sway path velocity as a function of the rehabilitation sessions. The mean values decreased from 33.4 ± 25.1 at the first VR session to 0.70 ± 17.0 at the fifth VR session (*p* < 0.01). The results attest to a better postural control with a reduced role of visual cues during the time course of posture normalization, and to significantly reduced energy spent by the patients to stand quietly.

**Table 2:** Modifications of the Romberg ratio assessed on the sway path velocity.

Romberg ratio (sway path velocity)	Mean (mm/s) (±SD)	*p* level
Session 1	33.4 ± 25.1	0.03
Session 2	14.4 ± 19.5	
Session 3	16.3 ± 18.9	0.04
Session 4	3.1 ± 20.7	
Session 5	0.7 ± 17.0	

The velocity of the center of foot pressure was recorded in the eyes open (EO) and eyes closed. (EC) conditions. The Romberg ratio (RR) was defined as following: RR = [(EC score − EO score)/(EC score + EO score) × 100]. Mean values (±SD) are given as a function of the rehabilitation sessions. The table shows significantly reduced values as early as the second vestibular rehabilitation session (*p* < 0.03), and more reduced scores later on (*p* < 0.04 between the third and the fourth session). Sway path velocity being a good estimate of the energy spent by the patients to keep balance, the data point to a better posture control over time with vestibular rehabilitation.

## 4 Discussion

Taken together, our data confirmed on a broader sample of AUVP patients the asymmetrical SVV bias we have previously observed (Haijoub and Lacour, 2024). Significantly higher estimates were found for ipsilateral laser line orientation and 30° preset angle compared to contralateral line orientation and 15° preset angle (retrospective study 1). They showed on the other hand that SVV normalization was significantly more quickly restored in AUVP patients rehabilitated on a base of support tilted to the hypofunction side compared to those with spontaneous normalization in absence of VR (prospective study 2). A close temporal correlation was also observed between SVV normalisation and posture normalisation.

### 4.1 Asymmetry of the SVV estimates

The SVV is elaborated in the brain on the basis of multisensory mechanisms including the integration of visual, vestibular and proprioceptive inputs (see introduction) that also contribute to recovery. SVV offset in AUVP patients is the result of vestibular tone imbalance in the roll plane. Unilateral peripheral vestibular damage causes changes in the graviceptive pathways conveying input from the otolith organs, at the origin of the so-called ocular tilt reaction. This eye-head synkinesis in the roll plane includes conjugate ocular torsion, skew deviation, head and body tilt, and SVV offset to the hypofunction side (Halmagyi et al., 1979; Curthoys and Halmagyi, 2014; Curthoys et al., 1991). There is a broad consensus to consider the SVV bias after unilateral vestibular loss as resulting from the imbalanced otolith afferent input to the vestibular nuclei (VN), and SVV normalisation as being due to a central compensation rebalancing the VN neural activity on both sides (see Lacour et al., 2015 for review). Arguments in favour of this recovery mechanism are first the similar temporal patterns of normalization for ocular cyclotorsion, head tilt and SVV reported in unilateral vestibular neurectomized patients (Redon et al., 2010), and second the recovery of electrophysiological homeostasis in the bilateral VN observed in animal models (Lacour et al., 2015).

The SVV bias to the hypofunction side in vestibular pathology has been attributed to the eye cyclotorsion (Curthoys et al., 1991) on the basis of the close temporal correlation between these two parameters. However, lesions of the thalamocortical vestibular pathways (Brandt et al., 1994), cerebral hemispheric stroke (Yelnick et al., 2002), and BPPV patients (Haijoub and Lacour, 2024) showed SVV bias without ocular torsion. Moreover, a dissociation between SVV tilt and ocular torsion was found in AUVP patients (Faralli et al., 2021), suggesting that these two signs of otolith dysfunction are only partially linked each other and share distinct re-balancing circuits. Finally, Dieterich and Brandt (1993) and more recent works clearly pointed to the lack of causal relationship between eye cyclotorsion and SVV (Kheradmand and Winnick, 2017; Kheradmand et al., 2016; Otero-Millan and Kheradmand, 2016).

SVV offset after unilateral peripheral vestibular damage has been recently modeled and two mechanisms were proposed (Glasauer et al., 2018). One is the otolith model in which application of Ewald’s law to the sensory cells with opposite morphological polarization vectors on both sides of the striola predicts an excitatory effect greater than the inhibitory effect during tilt or translation. With the utricular macula intact on both sides the otolith input is balanced and the SVV estimate is not biased. After unilateral peripheral vestibular loss the otolith input from the intact utricular macula remains unbalanced and would cause the SVV offset. This model fits the experimental data recorded in subjects with the head upright, that is, the patient’s head position during our SVV protocol. The otolith model remains however insufficient to explain the SVV offset when head/body was tilted in the roll plane, and the authors proposed a second model based on the effect of a torsional semicircular canal bias on the central gravity estimator.

Several hypotheses can account for the asymmetrical SVV bias and the preset angle amplitude effect. Whatever the preset line orientation, ipsilateral vs. contralateral with respect to the hypofunction side, the SVV bias was always towards the side of vestibular loss. This finding fits the otolith model of unbalanced otolith inputs in the graviceptive pathways from the periphery to the parieto-insular vestibular cortex. Bias in upright estimate due to body orientation in space have been described in healthy participants a long time ago as the Müller effect (or E-effect: Müller, 1916) and Aubert effect (A-effect: Aubert, 1861) for small (<60°) and larger (>60°) body tilts, respectively. The E-effect corresponds to an over-estimation of the perceived vertical resulting from an increased utricular weight. This E-effect was suppressed in patients with vestibular loss and replaced by the Aubert effect (Graybiel et al., 1968), a finding consistent with the reduced weight of head position (otolith) signals in our AUVP patients who underestimatd upright orientation. This change can explain the higher SVV bias for line orientation towards the side of vestibular loss. Systematic errors in the perceived upright orientation were also seen with lateral head or whole body tilts. Eventhough the SVV test was performed with the head upright, the central representation of verticality without orienting visual cues was biased to the side of vestibular lesion in our patients, a factor that can also contribute to the SVV bias asymmetry.

There is a strong effect of orienting visual cues on upright perception (cf. Figure 3) that has been evidenced in healthy and vestibular loss patients with the tilted room and the rod-and frame-test. Participants showed both postural body tilts towards the room or frame orientation, and SVV bias in the same direction. Similar effects on the perceived vertical were found with a simple line instead of a tilted square (Li and Matin, 2005). The SVV was biased by the orientation and the amplitude of the line orientation relative to gravity. Line orientations away from the gravitational vertical represent an attractor bias towards the line orientation, the weight of which depends on the amplitude of the preset angle. Higher SVV bias at 30° compared to 15° can be explained by this attractor bias.

The visual “entrainment” effect described by Mezey et al. (2004) cannot be responsible for the SVV asymmetry. This ocular torsion induced by the laser line rotation has a low gain in healthy participants and, if active in our AUVP patients, it should reduce the SVV offset. Our results showed opposite effects with a SVV bias significantly increased with preset angles on the hypofunction side and the laser line moving to the intact side. The contribution of neck muscle proprioception to SVV asymmetry is another hypothesis even though the role of neck afferents to compensate unilateral vestibular deficits remains open to debate. In the monkey model, however, the weight of neck muscle proprioception was increased as early as 1 week after unilateral vestibular lesion (Sadeghi et al., 2018). The imbalanced tonic otolithic input projecting to the neck motor neurons via the vestibulospinal pathways, and to the parieto-insular cortex via the vestibulo-thalamo-cortical pathways, could also be involved in the SVV asymmetry. A right hemispheric dominance has been reported in right-handed subjects (Dieterich et al., 2003). In a large sample of AUVP patients, Toupet et al. (2014) showed a slower SVV normalization in patients with right compared to left vestibular deficits. Asymmetry of otolith inputs at the cortical level could create the SVV asymmetry (Dieterich and Brandt, 2019). Our data also showed a greater SVV bias in the patients with right hypofunction, but our small sample findings need to be confirmed on a wider population.

### 4.2 Normalization of the subjective visual vertical

#### 4.2.1 Spontaneous normalization of the SVV

Spontaneous normalization of the SVV is described in the literature as a compensatory process closely related to the recovery of balanced neural activity in the vestibular nuclei (VN) on both sides and the restoration of VN electrophysiological homeostasis. This mechanism is also evoked for the normalization of spontaneous nystagmus (SN) over time (Lacour et al., 2023), and one common factor is the long time period required for SVV and SN normalization. The literature indicates that the static vestibular deficits (ocular tilt reaction) need at least 3 months to be fully compensated (Redon et al., 2010; Curthoys et al., 1991; Lopez et al., 2005), and even more for SVV normalization (6 months to 1 year: Böhmer and Rickenmann, 1995).

#### 4.2.2 Normalization of the SVV with the tilted support protocol

Our data showed a very fast SVV normalization in our AUVP patients rehabilitated with the tilted support protocol. Only 3 to 5 VR sessions, depending on the patients, were enough to recover a non pathologic SVV. By contrast, AUVP patients without VR and tested at the same time period after symptoms onset (1 month) still showed significantly higher and pathologic SVV bias.

Head-based graviceptive signals constitute the predominant input for internal estimates of visual vertical with head upright (Clemens et al., 2011; Tarnutzer et al., 2010). These signals combine with the head/body-fixed reference or idiotropic vector (Mittelstaedt, 1983) to elaborate an internal representation of the gravitational vertical. According to Mittelstaedt (1983), the brain uses an internal signal, an egocentric reference also called postural vertical or Z body axis vector in the literature, as an added signal to the otolith input to determine upright orientation. This idiotropic vector is responsible for the Aubert effect at large body tilts, and it reduces the upright perception distorsions at small body tilts. Based on such multimodal dependency, the SVV is altered but does not totally break down with impaired otolith organs. When the head-in space information from the otoliths is absent or distorted, an estimate of head orientation in space can be restored via other sensory pathways. The rationale of the tilted support protocol was to modify the patient’s body posture on the opposite side with respect to the SVV offset in order to increase the weight of the body sensors and to re-build a correct perception of verticality restoring the orientation constancy. As the otolith contribution becomes noisy because of asymmetrical inputs, the remaining sensory modalities are reweighted in proportion to their reliability (Angelaki and Cullen, 2008). A shift to a reference frame based on body in space orientation, that is, on an egocentric reference frame, was likely used to recalibrate the perception of verticality and to normalize the SVV. This hypothesis is supported by the concomitant symmetrization of both the CoP and the body weight distribution, suggesting a prominent role of the idiotropic vector over time.

Whereas CoP and body weight distribution were shifted to the hypofunction side during the first two VR sessions in the absence of orienting visual cues, they were shifted to the intact side with vision. Figures 3A,B, 4 illustrate this behavior that corroborates previous data on head and trunk orientation (Borel et al., 2008; Lopez et al., 2007a) and walking trajectory (Borel et al., 2004) in unilateral vestibular neurectomized patients. A contralateral shift of body weight was also reported in the first days after labyrinthectomy in the rat model in the light (Tighilet et al., 2017). Unfortunately the authors did not record the rat’s body weight distribution without vision, and they assimilated the contralateral shift to a behavioral strategy to avoid falling. Our data in AUVP patients indicate that the most likely hypothesis is a sensory strategy based on orienting visual cues, a major input to correct head and body orientation in space early after symptoms onset (Lopez et al., 2008). The early contribution of vision to posture control declines later on in our study as shown by the decreased Romberg quotient calculated on the CoP sway path, and the reduction of the sway velocity indicates a better postural performance with less energy spent to keep balance. VR on the tilted support likely increases the weight of the body sensors at later stages of the compensation process.

#### 4.2.3 Rehabilitation of the subjective visual vertical

Our study showed that rehabilitation of AUVP patients with the tilted support protocol has positive outcomes on both SVV and posture normalization. It is the only one in the literature to describe the recovery time course of SVV normalization, and to propose a clinical protocol that can be used as an additional tool in vestibular rehabilitation to recover a stable spatial perception necessary for sensorimotor planning in everyday life.

Tilting the base of support is not the only way to rapidly recover the perception of verticality. Faster SVV normalization compared to spontaneous recovery in absence of VR has been observed with VR protocols as different as gaze stabilization exercises and the unidirectional rotation paradigm (Lacour et al., 2021). The objectives and expected effects of these different protocols, as well as the very characteristics of these rehabilitation methods, however differ very greatly. Gaze exercises are aimed at recovering gaze stabilization abilities either by restoring the semicircular canal dynamics (vestibulo-ocular reflex gain) or by promoting new behavioral strategies (saccadic substitution). The dynamic visual acuity training, for instance, is based on active and dynamic training of the patients to restore their gaze stabilization using adaptive mechanisms. On the other hand, the rotatory chair protocol is a passive stimulation consisting of repeated whole body rotation to the weaker side in order to reduce the spontaneous nystagmus and to decrease the asymmetrical vestibulo-ocular responses. The protocol is based on both habituation of the vestibular response on the healthy side, and possible stimulation of remaining vestibular afferent fibers on the hypofunction side when VR is performed early after symptoms onset, two plasticity mechanisms restoring the electrophysiological homeostasis in the vestibular nuclei. The tilted support is a mixed protocol which includes a static stimulation inducing dynamic postural corrections that depend on the environmental context (with or without vision, normal or altered somatosensory information). The goal here is the rehabilitation of gravity perception and orientation constancy, a process very likely requiring changes in the spatial reference frames at high cortical levels.

We recently published a mini review showing that different VR protocols can really interfere with the plasticity mechanisms participating in the recovery of the static vestibular deficits (Lacour and Haijoub, 2024). These different rehabilitation tools can speed up SVV normalization in AUVP patients because regain of electrophysiological homeostasis in the VN, a key mechanism to compensate the static vestibular deficits, is achieved through various brain plasticity mechanisms, ranging from cellular and molecular events to sensory and behavioral substitution processes with which training and rehabilitation interact (see Lacour and Bernard-Demanze, 2015 for review). Brain plasticity-based therapeutics are the way to normalize the defective vestibular functions (Merzenich et al., 2014). That includes the SVV, a perceptive symptom more dependent on egocentric (proprioception), allocentric (vision), and geocentric (otolith) spatial reference frames than simple vestibular reflexes. Studies with different VR protocols and follow-up of the SVV bias as a function of the VR sessions are further required.

## 5 Conclusions and limitations of the study

We recommend routinely considering the SVV asymmetry in the clinic and to individually assess the ipsilateral and contralateral SVV for a better assessment of otolith organs imbalance that can trigger chronic instability and dizziness. The VR protocol consisting in tilting the base of support to the hypofunction side, that is, to the side of the SVV bias, is an additional means in the physiotherapist’s toolbox to rapidly normalize the SVV, and it can be applied to elderly patients with a tendency to fall backwards. It is not a magic tool but a relatively simple way to rapidly recover a good posture control and an adaquate perception of orientation in space.

Further investigations should more precisely examine how the sensory signals are reweighted and how different VR protocols can modify the reference frames to recover spatial orientation and posture control after peripheral vestibular loss in different vestibular pathologies. Such investigations should orient the physiotherapists towards the best protocol for their vertigo and dizzinessy patients.

## Data Availability

The raw data supporting the conclusions of this article will be made available by the authors, without undue reservation.
